# Supplementary material for: A Global Screen for Assembly State Changes of the Mitotic Proteome by SEC-SWATH-MS
Source: Cell Syst. 2020 Feb 26;10(2):133–155.e6. doi: 10.1016/j.cels.2020.01.001 (PMC7042714; doi:10.1016/j.cels.2020.01.001)

Q9P1Z2 | CACO1_HUMAN | CALCOCO1 KIAA1536 PP13275 UNQ2436/PRO4996
Monomer MW [kDa]: 77.336 Monomer expected elution fraction: 42

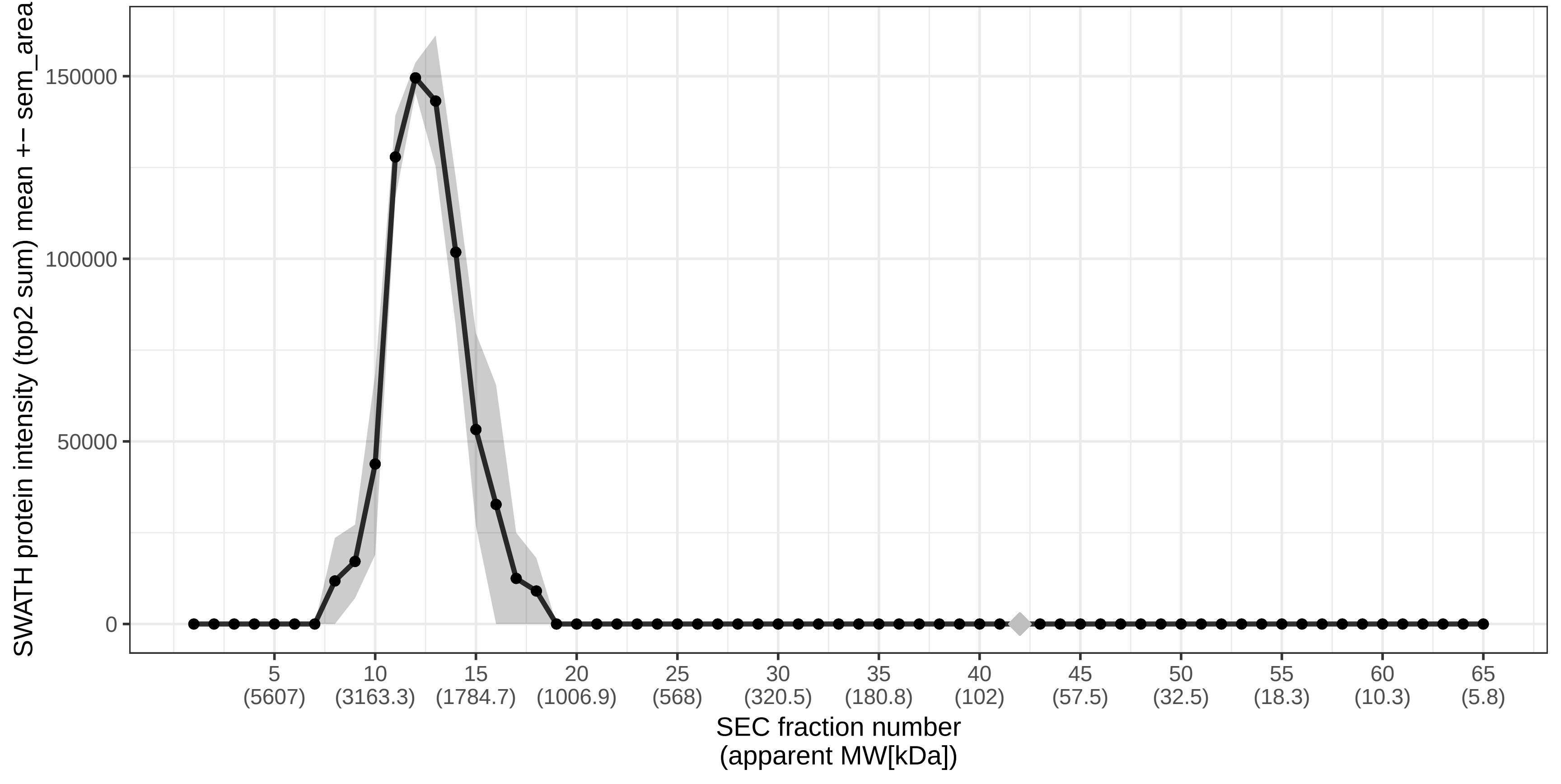

Supplement: Data S1. SEC-SWATH-MS Protein Chromatograms, Related to Figure 1 [file mmc6.zip › SECchrom_Q9P1Z2_CACO1_HUMAN_CALCOCO1_KIAA1536_PP13275_UNQ24.pdf]
